# Efficient Cow Body Condition Scoring Using BCS-YOLO: A Lightweight, Knowledge Distillation-Based Method

**DOI:** 10.3390/ani14243668

**Published:** 2024-12-19

**Authors:** Zhiqiang Zheng, Zhuangzhuang Wang, Zhi Weng

**Affiliations:** 1 College of Electronic Information Engineering, Inner Mongolia University, Hohhot 010021, China; zqzheng@imu.edu.cn (Z.Z.); 32356154@mail.imu.edu.cn (Z.W.); 2 State Key Laboratory of Reproductive Regulation & Breeding of Grassland Livestock, Hohhot 010021, China; 3 Research Base for Dairy Farming Engineering and Full Mechanization of Equipment, Inner Mongolia Agricultural University, Ministry of Agriculture and Rural Affairs, Hohhot 010018, China

**Keywords:** cow body condition scoring, lightweight design, YOLOv8, SSLDH, knowledge distillation

## Abstract

Monitoring the body condition of dairy cows is essential for ensuring their health and productivity, but traditional BCS methods—relying on visual or tactile assessments by skilled personnel—are subjective, labor-intensive, and impractical for large-scale farms. To overcome these limitations, we present BCS-YOLO, a lightweight and automated BCS framework built on YOLOv8, which enables consistent, accurate scoring under complex conditions with minimal computational resources. BCS-YOLO integrates the Star-EMA module and the Star Shared Lightweight Detection Head (SSLDH) to enhance the detection accuracy and reduce model complexity. The Star-EMA module employs multi-scale attention mechanisms that balance spatial and semantic features, optimizing feature representation for cow hindquarters in cluttered farm environments. SSLDH further simplifies the detection head, making BCS-YOLO viable for deployment in resource-limited scenarios. Additionally, channel-based knowledge distillation generates soft probability maps focusing on key body regions, facilitating effective knowledge transfer and enhancing performance. The results on a public cow image dataset show that BCS-YOLO reduces the model size by 33% and improves the mean average precision (mAP) by 9.4%. These advances make BCS-YOLO a robust, non-invasive tool for consistent and accurate BCS in large-scale farming, supporting sustainable livestock management, reducing labor costs, enhancing animal welfare, and boosting productivity.

## Simple Summary

Automated systems are transforming livestock management by addressing the limitations of traditional body condition scoring (BCS), which is labor-intensive and impractical for large-scale farms. We developed BCS-YOLO, an enhanced YOLOv8-based framework tailored to automated, non-contact BCS. The model achieves a 33% reduction in size and a 9.4% improvement in scoring accuracy by focusing on key anatomical features like the back and tail. Its compact design ensures real-time scoring with minimal computational requirements, making it suitable for resource-constrained environments. BCS-YOLO not only reduces labor costs and supports efficient health monitoring, but also promotes sustainable livestock management and intelligent agriculture.

## 1. Introduction

BCS is not only a tool for assessing the nutritional status of dairy cows, but is also a key indicator reflecting animal health and welfare. Clinically, BCS is considered the fifth vital sign for evaluating dairy cow health, providing valuable clues for the early detection of diseases. This is crucial for ensuring the health of the herd and preventing the spread of disease. Additionally, BCS plays an irreplaceable role in production management, enabling farmers to accurately assess the nutritional condition of their cows and, in turn, adjust feeding strategies. This helps optimize breeding and feed plans effectively. It is conducted on a 5-point scale with 0.25-point increments, where a score of 1 reflects extreme emaciation and 5 indicates severe obesity [1]. Maintaining an appropriate BCS is essential for the health, reproduction, and productive lifespan of high-yielding dairy cows, particularly during early lactation when energy demands peak. Excessive body condition loss during this phase can result in ketosis, a fatty liver, and reproductive issues such as longer estrus intervals and higher breeding failure rates [1].

Traditional BCS methods rely on visual observation and palpation, where evaluators inspect the back and pelvic regions, particularly assessing the fat cover at the tailhead, feeling the thickness of the fat layer at the loin and transverse processes, and observing the prominence of the pelvis [1]. However, this manual method is time-consuming and subject to subjective judgment, leading to inconsistent scoring results. Frequent handling may also stress the cows, increasing the risk of aggressive behavior and impacting the safety and efficiency of the scoring process. These challenges have limited the widespread application of BCS [2]. A survey indicated that only 36% of the surveyed herds implemented BCS in their management practices [3]. Despite the significant value of BCS, its practical use has not been widespread due to the limitations of traditional methods.

Therefore, more user-friendly machine scoring systems have begun to emerge. For instance, Halachmi et al. (2013) [4] utilized thermal imaging technology to score dairy cows; however, variations in hair thickness masked changes in the temperature of the fat layer, undermining the initial concept based on fat’s insulating properties and reducing the scoring effectiveness. Janzekovic et al. (2015) [5] achieved extremely high BCS accuracy by using an ultrasound measurement device to scan specific areas of a cow’s body, but the experiment required the cows to be individually placed in self-locking feed stalls, adding a layer of complexity.

With the advent of computer technology and artificial intelligence, lightweight visual analysis has gradually been applied in the agricultural sector. The field of visual analysis is divided into three-dimensional (3D) and two-dimensional (2D) approaches.

In terms of 3D vision, Alvarez et al. (2018) [6] developed an automated BCS evaluation system based on convolutional neural networks (CNNs). The system utilizes depth images and adopts a compact CNN architecture, incorporating specialized modules and strategies to reduce parameter size. When the scoring error margin is 0.5, the system achieves a relatively high level of accuracy, making it suitable for deployment in resource-constrained GPU environments. Yukun et al. (2019) [7] developed a convolutional neural network based on depth, grayscale, and phase consistency channels for individual identification and BCS evaluation. By employing a lightweight model along with data augmentation and optimization techniques, the system achieved a certain degree of parameter reduction while maintaining both efficiency and accuracy. Wang et al. (2024) [8] employed the EfficientBCS model combined with a depth RGB camera for the BCS scoring of dairy cows. To achieve a lightweight design, EfficientBCS integrated attention mechanisms with a lightweight model, keeping the model parameters under 85M. This system achieved a predictive accuracy of 46.2% within a zero-error scoring range.

In terms of 2D imaging, Huang et al. (2019) [9] introduced an improved SSD algorithm, achieving model lightweighting by replacing network connections and transforming convolution methods, ultimately keeping the model size at 23.1 MB while enhancing performance. Feng et al. (2024) [10] proposed a lightweight model that integrated attention modules to enhance focus on key features and replaced activation functions to reduce computational resource consumption, achieving a lightweight design.

Traditional 3D vision-based body condition scoring not only requires the complex 3D processing of dairy cow data, but also relies on high-cost data collection equipment. While 2D image acquisition reduces equipment costs, highly complex artificial intelligence neural networks consume significant computational resources, which hampers the practicality of image processing in real-world production.

Although advances in neural network lightweighting technology have led to breakthroughs in reducing parameter counts and computational resource consumption, new methods need to be explored to achieve higher accuracy and performance with even fewer parameters to meet the efficiency demands of real-world production [11]. Therefore, achieving efficient body condition scoring detection for dairy cows in practical production environments remains challenging and requires further exploration.

Therefore, this paper proposes a dairy cow body condition scoring algorithm, optimized based on the YOLOv8 framework—BCS-YOLO. This algorithm uses a 2D camera installed above the alleyways in dairy farms to capture tail images of cows, enabling non-contact body condition scoring. This method significantly reduces the subjective bias and inefficiency associated with traditional scoring techniques and introduces a new approach to model lightweighting. Although some established animal body condition scoring algorithms exist, there has been no experimental validation of the latest publicly available dataset used in this study. Consequently, this research optimizes existing models to enhance the detection performance and deployment efficiency, providing an efficient, scalable solution for the field. This advancement supports the development of automated body condition evaluation technology and lays a foundation for future research and applications. The main methods of this study include the following:

(1) The original C2f module of YOLOv8 was optimized by integrating an efficient multi-scale attention mechanism [12] into the StarNet [13] network structure, resulting in the proposed C2f-Star-EMA module. This optimization expanded the feature space dimensions of the network and balanced the spatial–semantic distribution within feature groups, allowing the network to better focus on key areas.

(2) The detection head module was replaced with the SSLDH module proposed in this paper, enabling the model to adapt to environments with limited computational resources in a more lightweight form.

(3) A targeted approach using feature distillation based on CWD (Channel-wise Knowledge Distillation) [14] was adopted, significantly improving the detection accuracy and further enhancing model performance.

Finally, as a result of the aforementioned optimization strategies, the model’s weight size was reduced by 33%, and the mAP at an Intersection over Union (IoU) threshold of 50% was improved by 9.4%. These results underscore the effectiveness of our efforts in model lightweighting and validate the utility of these optimization approaches in enhancing detection performance. This advancement provides substantial support for the broader application and adoption of body condition scoring detection models for dairy cows.

## 2. Materials and Methods

### 2.1. Materials

#### 2.1.1. Dataset Information

**Figure 1:**
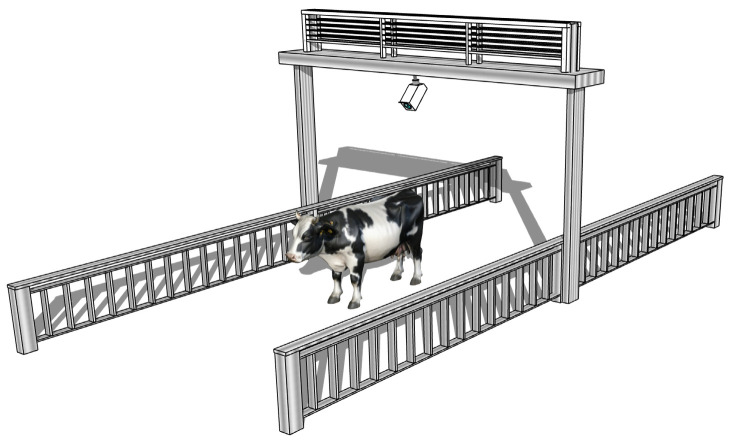
Schematic diagram of the cow data collection setup.

This study utilized a publicly available dairy cow body condition scoring dataset [15] for object detection analysis. Data were collected between May 2018 and July 2023 from three locations: Huahao Ecological Farming Co., Ltd. in Lu’an, Anhui Province, China; Xuyi Weigang Pasture Co., Ltd. in Huai’an, Jiangsu Province, China; and Xinmuwang Pasture Co., Ltd. in Wuwei, Gansu Province, China. The dataset does not include information on cow breed or age. Data were captured using Hikvision starlight network cameras (manufactured in Hangzhou, China) with a resolution of 1297 × 720 pixels. The cameras were mounted 2.4 m above the ground in milking lanes, positioned to provide an overhead view of the cow tailhead region. Videos were recorded at an original frame rate of 25 frames per second, with the dataset constructed by extracting one frame every three frames. Figure 1 illustrates the video data acquisition setup, detailing the camera positions and sampling scenarios as cows passed through the alleyway.

To ensure diversity within the dataset, the collection process encompassed various lighting conditions, complex backgrounds, and a range of cow postures.

#### 2.1.2. Data Screening and Partitioning

The public dataset used in this study contains 53,566 images of dairy cow BCS, all annotated and scored by a professional team of veterinarians. Each image includes a bounding box around the cow’s tail and an associated BCS label, providing precise training data for the model. In the original dataset, the distribution of images per BCS category was as follows: 7536 images for BCS 3.25, 13,256 images for BCS 3.5, 14,255 images for BCS 3.75, 12,556 images for BCS 4.0, and 5963 images for BCS 4.25.

**Figure 2:**
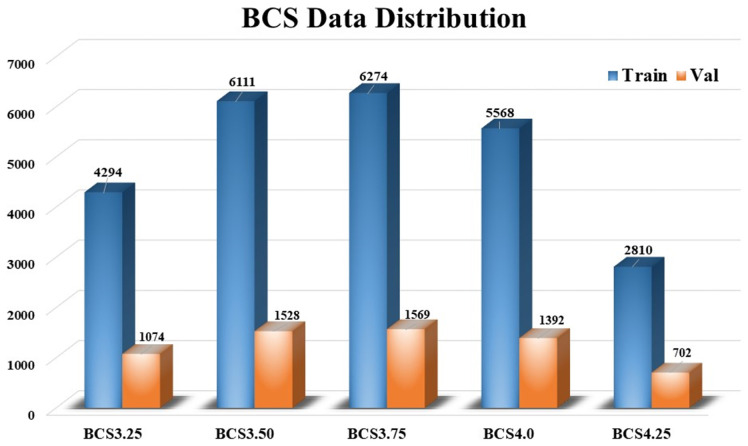
BCS training and validation data distribution across different scores.

To enhance the model’s generalization ability and reduce the risk of data leakage, the original dataset was manually re-screened to remove highly similar images. The screening process first involved grouping images based on their BCS category and the unique identifier (ID) of the cow, ensuring the systematic organization of images for each individual cow. Within each group, visually similar images with minimal frame-to-frame content differences were manually filtered out. Specifically, images were retained if they exhibited noticeable variations in posture, lighting conditions, or clarity, while redundant images were excluded, thereby increasing the diversity and representativeness of the dataset. After re-screening and resampling, the distribution of images per BCS category was adjusted to 5368 images for BCS 3.25, 7639 images for BCS 3.5, 7843 images for BCS 3.75, 6960 images for BCS 4.0, and 3512 images for BCS 4.25, totaling 31,322 images. To ensure data balance and effective training, each category was split into a training and test set at a ratio of 8:2. This division method enables a more objective evaluation of the model’s performance (Figure 2).

### 2.2. Cow BCS Object Detection

#### 2.2.1. YOLOv8

The YOLOv8 algorithm, released by Ultralytics, is a popular model supporting various vision tasks. Its core structure is similar to YOLOv5 but incorporates the C2f module to enhance feature extraction. The C2f module consists of two convolutional layers, which help in extracting features of different levels and abstractions from the input data, thereby improving detection performance [16].

The YOLOv8 network architecture is centered on a single-input design. The workflow begins with reading image data, followed by a series of data augmentation steps, including random scaling, cropping, flipping, and color adjustment. These augmentations increase data diversity, enhancing the model’s generalization ability. The image is then resized to a fixed input size and normalized by pixel values, typically mapped to the range [0, 1], to ensure training stability and accelerate convergence. During the feature extraction phase, the image data are processed through a well-designed convolutional kernel along with batch normalization techniques, which helps speed up training and stabilize network performance. YOLOv8’s uniqueness lies in its enhanced feature extraction architecture, which focuses on fusing feature information across different scales [17]. The model efficiently processes targets across small (20 × 20), medium (40 × 40), and large (80 × 80) scales through dedicated structural optimization to meet the detection needs of various object sizes and details. Throughout the processing flow, multi-scale features are skillfully integrated and evaluated by the core network components. Ultimately, the comprehensive detection results not only reflect YOLOv8’s superior analytical capabilities in target detection tasks, but also highlight its performance in multi-scale information fusion.

#### 2.2.2. StarNet

In BCS tasks, accuracy and efficiency are crucial. Traditional Convolutional Neural Networks often encounter limitations when handling complex feature structures, particularly when dealing with multi-scale and high-dimensional input data. Additionally, conventional feature fusion methods are often inadequate in capturing the complexity of such data [18]. Therefore, selecting a network architecture that can effectively enhance feature representation is vital for improving the accuracy of BCS tasks.

The StarNet architecture, proposed by Xu Ma et al. at the 2024 CVPR conference, introduces an innovative “star operation” that fuses linearly transformed features. Unlike traditional additive fusion, the star operation uses element-wise multiplication to map the input data into a high-dimensional, non-linear feature space, significantly enhancing the network’s feature representation capacity. This high-dimensional feature space enables the network to better capture the intricate structure of the data, making it particularly well suited for BCS tasks involving multi-scale inputs and complex backgrounds.

StarNet tackles the specific challenges of BCS tasks by enhancing the combination of features and improving both local feature expression and global feature integration. This is especially important because the model needs to not only distinguish between different body conditions of cows, but also ensure accurate classification in real-world, challenging scenarios. Furthermore, StarNet demonstrates stronger robustness when handling multi-scale inputs and complex backgrounds, maintaining a high detection accuracy even when faced with dynamic blur. These improvements make StarNet a more reliable solution for BCS tasks, especially when compared to traditional CNN architectures, which often struggle with such complex real-world conditions.

**Figure 3:**
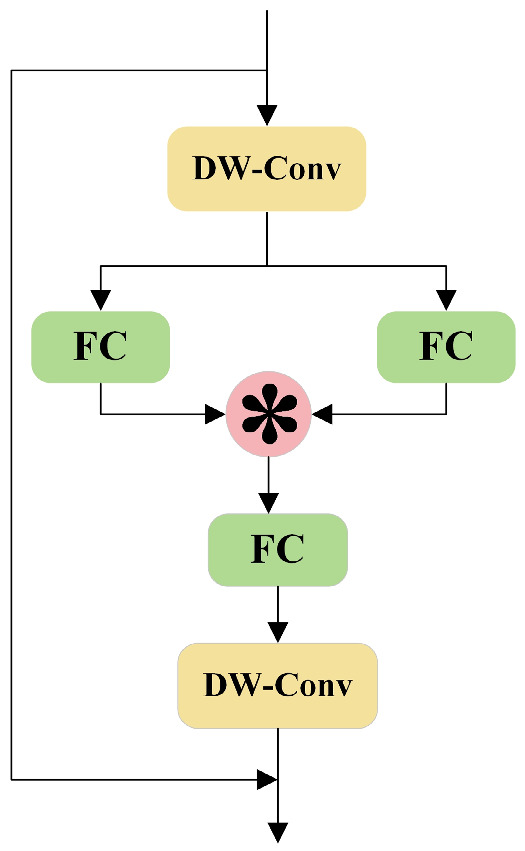
Star network structure, where FC is the fully connected layer and DW-Conv is the deeply separable convolutional.

The StarNet network structure is shown in Figure 3. The star operation proposed in this paper is a method used to fuse two linear transformed features by element-level multiplication. This operation is capable of mapping the input data into a high-dimensional, non-linear feature space, thus enhancing the feature representation capability of the neural network. The star operation is usually expressed as an element-wise multiplication of two linearly transformed features and is usually written as $\left(W_{1}^{T}X+B_{1}\right)*\left(W_{2}^{T}X+B_{2}\right)$, where ∗ denotes the element-wise multiplication; for simplicity of representation, we combine the weight matrix and the bias term into a single entity, denoted by $W=\left[\begin{array}{c} W\\ B \end{array}\right]$. Correspondingly, the input is also denoted by $X=\left[\begin{array}{c} X\\ 1 \end{array}\right]$. We consider the case of a single output channel and a single element input $w_{1},w_{2},x\in R^{\left(d+1\right)\times 1}$, where *d* is the number of input channels and the star operation can be expressed as $w_{1}^{T}x*w_{2}^{T}x$. Expanding the star operation, we obtain the following:

(1) $$w_{1}^{T}x*w_{2}^{T}x,$$

(2) $$=\left(\sum_{i=1}^{d+1}w_{1}^{i}\;x^{i}\right)*\left(\sum_{j=1}^{d+1}w_{2}^{j}\;x^{j}\right),$$

(3) $$=\sum_{i=1}^{d+1}\sum_{j=1}^{d+1}w_{1}^{i}w_{2}^{j}x^{i}x^{j},$$

To simplify the representation of each term, we introduce the coefficients $\alpha \left(i,j\right)$, with the definition of these coefficients taking into account two scenarios: when i=j, it is the product of the elements in the same position, $\alpha \left(i,j\right)=w_{1}^{i}w_{2}^{j}$. When i≠j, $\alpha \left(i,j\right)=w_{1}^{i}w_{2}^{j}+w_{1}^{j}w_{2}^{i}$. An important property of the star operation is its ability to map inputs to high-dimensional, nonlinear feature spaces. Specifically, the star operation maps a d-dimensional space to an implicit $\frac{\left(d+2\right)\left(d+1\right)}{2}$ dimensional feature space, which implies the implementation of high-dimensional feature representation within a computationally efficient operation. For BCS tasks, this enhanced feature representation ability aids in more accurately identifying the body condition of cows, particularly when dealing with complex backgrounds or dynamic blur.

#### 2.2.3. EMA

In the task of BCS for dairy cows, image-based scoring methods inevitably face challenges such as fluctuations in image quality, dynamic blurring, and complex background interference, making it more difficult to accurately assess the cow’s body characteristics. Dynamic blurring and background interference often compromise the stability of the scores. Furthermore, completely integrating algorithms designed to address dynamic blurring can increase model complexity, making it difficult to meet the requirements of low-resource environments.

To overcome these challenges, the Efficient Attention Module proposed by Dalian Ouyang et al. at ICASSP 2023 offers an effective solution. This module employs a multi-scale attention mechanism that enables the model to focus on key regions of the cow’s body, suppress background interference, and improve the extraction of body condition information, thus alleviating the impact of dynamic blurring. Simultaneously, the EMA module reduces the parameter count and computational complexity, optimizing computational efficiency and alleviating the system’s computational burden, thereby addressing the lightweight requirements of current scoring systems.

As a result, EMA demonstrates unique advantages in improving scoring accuracy and robustness, particularly in challenging conditions involving dynamic blurring and background interference, providing more stable and precise results. EMA offers a more efficient, accurate, and lightweight solution for intelligent BCS. To fully exploit the advantages of EMA, this paper adopts this module, taking into account the specific characteristics of the BCS task; this is with the goal of enhancing feature map representation through a multi-scale attention mechanism, thereby optimizing feature extraction for the BCS task.

The specific principles of this module are as follows: First, the input feature map is $X\in {\mathrm{IR}}^{C\times H\times W}$. In the horizontal direction, global information is extracted using 1D global average pooling, which is calculated as follows:

(4) $$z_{g}^{H}\left(c,i\right)=\frac{1}{W}\sum_{j=1}^{W}x_{g}\left(c,i,j\right),$$

In this context, $x_{g}\left(c,i,j\right)$ represents the value of the *g*-th sub-feature map at channel *c*, height *i* and width *j*. This operation captures the long-range dependencies of the feature map at different horizontal locations via the global pooling of the width dimension along the horizontal direction. Similarly, in the vertical direction, global information is extracted by 1D global average pooling, as specified in Equation:

(5) $$z_{g}^{W}\left(c,j\right)=\frac{1}{H}\sum_{i=1}^{H}x_{g}\left(c,i,j\right),$$

Finally, the encoded features in the horizontal and vertical directions are stitched together and the final feature map is generated by 1 × 1 convolution:

(6) $$z_{g}=\sigma \left(W_{1}\left[z_{g}^{H}⊕z_{g}^{W}\right]\right),$$

where ⨁ denotes the feature splicing operation, $W_{1}$ is the 1 × 1 convolutional kernel, and σ is the Sigmoid activation function. Thus, the EMA module effectively fuses global information from various directions to produce feature maps with enhanced characterization capabilities. Additionally, to further improve feature representation, the EMA module combines the output feature maps from all sub-feature maps using a simple average pooling operation. This approach not only enhances the global representation and local detail capture of the feature maps, but also maintains low computational complexity.

**Figure 4:**
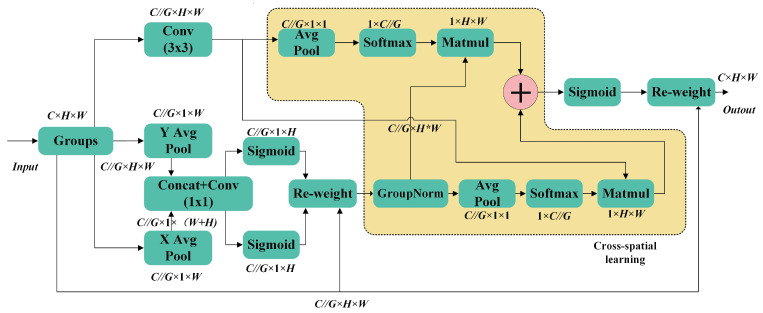
Architecture of the EMA Module, where ‘*G*’ denotes grouping, ‘*X* averaging pool’ denotes 1D horizontal global merging, and ‘*Y* averaging pool’ denotes 1D vertical global merging.

These features allow the EMA module to deliver more accurate and effective feature representations for practical applications such as cattle body condition scoring. Additionally, it demonstrates significant advantages in parametric and computational efficiency, particularly in handling complex backgrounds and multi-scale problems (Figure 4).

### 2.3. Optimization Strategy

According to Roseler et al. (1998) [19], when the BCS of dairy cows exceeds 3.75, an increase of 0.25 points can result in a reduction in dry matter intake (DMI) by approximately 1.5% to 2%. This indicates that even slight changes in BCS can significantly impact the nutritional intake and overall productivity of the cows. Additionally, a 0.25-point difference is generally considered insignificant and often relies on the subjective judgment of the scorer [20]. At the same time, there may be subjective differences in judgment among different veterinarians. Moreover, performing body condition scoring on large-scale farms requires a significant number of specialized veterinarians and a considerable amount of time. Additionally, the early detection of health issues in individual cows and timely intervention are crucial. Clearly, this is not the optimal solution. Therefore, ensuring the accuracy and convenience of the scoring system is crucial. To utilize the scoring system in a wider range of applications and employ it in a cost-effective, high-precision manner in resource-constrained environments, the lightweighting of the model becomes particularly important.

To address these challenges and needs, we developed a contactless cow body condition scoring model, BCS-YOLO, based on the YOLOv8 algorithmic framework, employing a multi-dimensional optimization strategy. Firstly, by optimizing the backbone layer, neck structure, and detection head, and utilizing scoring data from professional veterinarians for training, the model achieves enhanced objectivity while improving both the scoring accuracy and efficiency. We adopted a lightweight network structure design that effectively reduces the model size and computational resource requirements. This design facilitates the model’s application in environments with limited computational resources and supports the widespread adoption of intelligent body condition scoring for dairy cows.

#### 2.3.1. Proposing New Modules

To meet the specific demands of the BCS task, this paper proposes the C2f-Star-EMA module to balance the trade-off between model accuracy and size. Traditional scoring models typically have large parameter counts, making them challenging to deploy widely, while lightweight models may suffer from reduced accuracy. The C2f-Star-EMA module reduces the parameter count while enhancing accuracy, thus improving the deployment efficiency in practical applications.

This module focuses on three key aspects. First, by expanding the feature space dimensions and balancing the spatial semantic distribution within feature groups, C2f-Star-EMA significantly enhances the model’s ability to capture fine-grained features, particularly when processing images of cows with similar BCS ratings. This allows the model to more accurately focus on critical areas in the BCS task (such as the cow’s tailhead), thereby improving classification accuracy and stability.

Second, through network structure optimization, channel reduction, and lightweight design integration at critical layers, C2f-Star-EMA achieves model lightweighting while maintaining accuracy. This improvement not only lowers deployment costs but also strengthens the model’s adaptability and scalability for large datasets and complex tasks.

Third, the module greatly enhances model robustness. The star operation in StarNet maps inputs to a high-dimensional, nonlinear feature space, increasing the diversity of feature representations, while the multi-scale global attention mechanism in the EMA module further reinforces long-range dependency capture. This design enables the model to effectively handle motion blur and complex backgrounds in dynamic environments, ensuring stability and accuracy across diverse conditions.

In summary, the C2f-Star-EMA module’s improvements in accuracy, lightweighting, and robustness enhance the model’s adaptability for the BCS task, providing greater practicality and applicability in real-world scenarios.

#### 2.3.2. Propose New Detection Head

Achieving model lightweighting while maintaining high accuracy is crucial for widespread deployment in resource-constrained environments, particularly in applications like cattle BCS. However, the original YOLOv8 detection head faces limitations due to its structural design, leading to a high parameter count that consumes one-fifth of the total computation. This hinders the model’s lightweight deployment and its ability to run efficiently in scenarios with limited computational resources. To address these issues, this paper introduces the SSLDH module. Inspired by a star-shaped structure, the SSLDH module significantly reduces the parameter count through parameter sharing and is specifically designed for tasks requiring fine-grained feature capture, making it particularly suitable for BCS in cattle.

The SSLDH module first utilizes multi-scale input feature layers (P5, P4, P3) and adjusts the channel dimensions via GN_Conv 1x1 convolutional layers, which enhance feature expression while maintaining computational efficiency. Subsequently, features from different scales are integrated through a shared GN_Conv 3 × 3 convolutional layer, enabling the deep fusion of multi-scale information. Additionally, the merged features undergo further interaction through two fully connected (FC) layers, with an element-wise multiplication operation between them to enhance feature expression across multiple levels. Finally, the features are further refined through another shared GN_Conv 3 × 3 convolutional layer to produce more precise detection outputs. In the output layer, Conv_Reg and Conv_Cls convolutional modules generate bounding box regression and classification outputs, with a Scale layer to adjust for different detection needs.

Another notable feature of the SSLDH module is the integration of GroupNorm convolution (GN_Conv), a technique shown in FOCS (Feature-Oriented Convolutional Structures) [21] research to enhance localization and classification performance. GN _Conv divides the input tensor into groups based on channel count, with each group normalized independently, thus reducing the sensitivity to batch size, which is especially beneficial for small-batch or single-image inputs. Following normalization, features are further enhanced by the SiLU activation function, strengthening nonlinear expression and enabling the model to effectively distinguish fine-grained feature differences in complex BCS tasks. Moreover, the star-shaped structure of SSLDH projects input features into a high-dimensional space for richer detail capture, while computations remain in a lower-dimensional space. This design not only excels in information capture but also maintains computational efficiency, thereby significantly enhancing the network’s representational and generalization capabilities.

**Figure 5:**
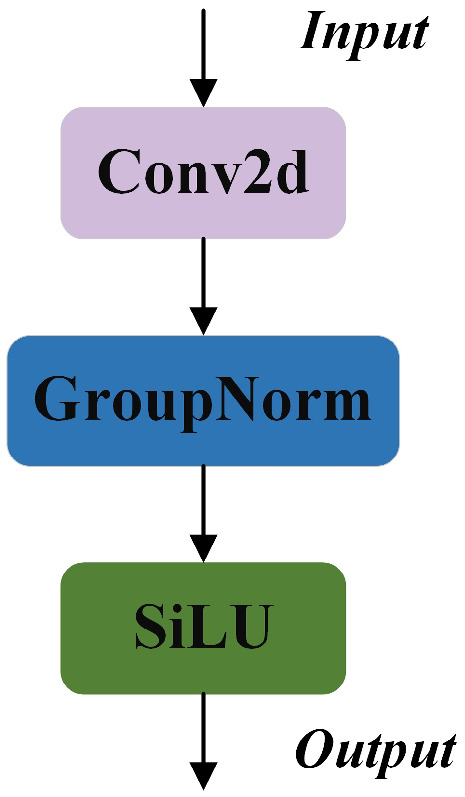
Architecture of the GN_Conv module.

In the context of cattle BCS, the core advantage of the SSLDH module lies in its lightweight parameter structure and comprehensive utilization of multi-scale features. The deep fusion and enhancement of multi-scale features allow the system to more accurately capture subtle differences between BCS categories, thus improving the scoring accuracy and reliability (Figure 5).

#### 2.3.3. CWD Feature-Based Knowledge Distillation

**Figure 6:**
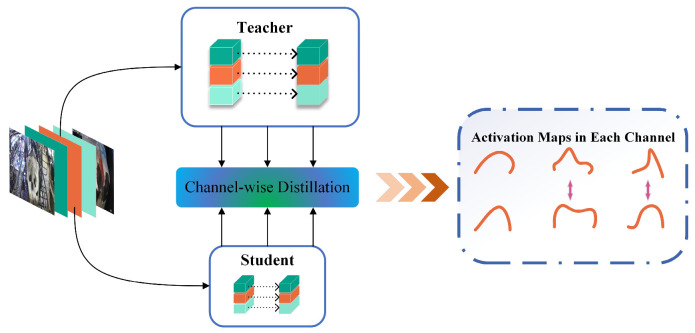
Structure of CWD feature-based knowledge distillation.

Traditional knowledge distillation methods usually align the feature maps of the teacher and student networks in the spatial domain by minimizing point-to-point or pairwise differences [22]. However, these approaches often fail to fully leverage the information within each channel, potentially overlooking the details essential for complex prediction tasks. In contrast, CWD generates a soft probability map by normalizing the activation maps of each channel, thus capturing the more nuanced information crucial for accurate predictions. This soft probability map emphasizes the most salient regions within each channel, enabling the model to focus more on areas with significant variations in tail tissue coverage when processing images of cow tails. These regions are crucial for accurate classification in the detection task. By minimizing the Kullback-Leibler (KL) [23] divergence between the channel-level probability maps of the teacher and student networks, knowledge transfer becomes more efficient. Specifically, given an input feature map (or logits map) of size *h_f_* × *w_f_* × *c* or *h_s_* × *w_s_* × *n*, where *h_f_* and *w_f_* (or *h_s_* and *w_s_*) are the height and width of the feature map (or logits map), respectively, c is the number of channels, and n is the number of categories, the channel-level probability distributions between the two networks are matched by employing a simple strategy of minimizing KL scatter. This approach not only significantly improves the performance of the student network, but also keeps the computational cost low and ultimately accomplishes model enrichment while maintaining model performance (Figure 6).

The left side illustrates the teacher–student strategy, leveraging feature and logits maps for channel-based knowledge distillation. The right side provides an intuitive graphical representation of the process.

#### 2.3.4. Improvement of Trunk Necks and Detection Heads

**Figure 7:**
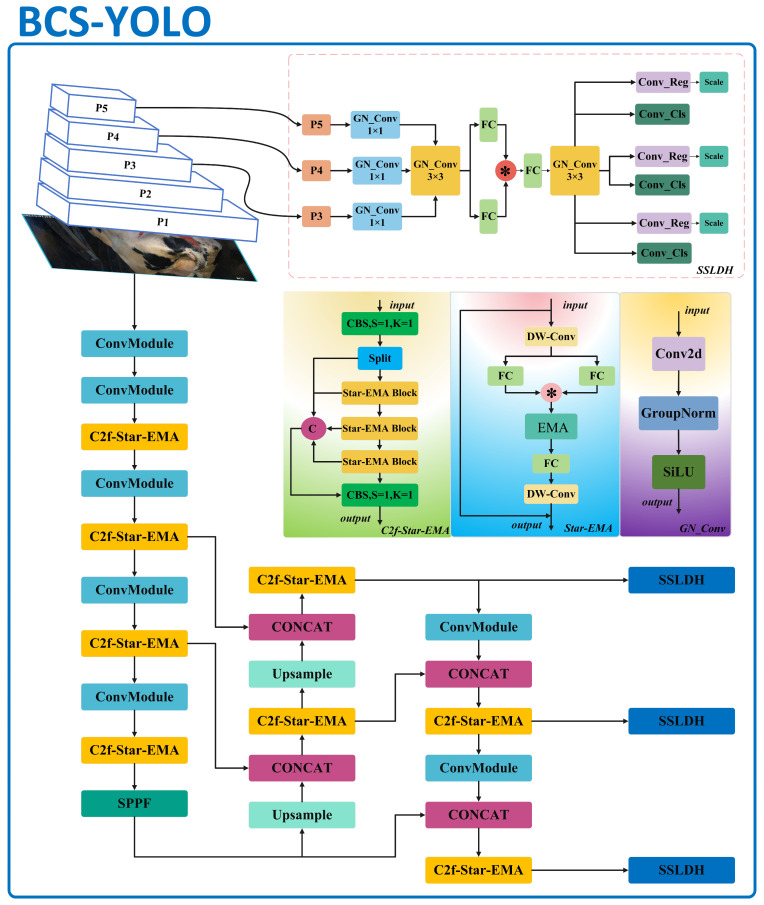
Architecture of the BCS-YOLO model.

The structure of the BCS-YOLO model is illustrated in Figure 7. Its core focus is on optimizing the Backbone, Neck, and Detection Head components for tasks such as cattle body condition scoring. Specifically, the C2f-Star-EAM module replaces the original C2f module in both the Backbone and Neck sections. This enhancement substantially improves the network’s accuracy, particularly in detecting critical areas, making it more sensitive and efficient. Additionally, the model substitutes the original Detection Head with a more lightweight SSLDH module. This change enhances its multi-scale sensing capabilities, further reduces the model size, and improves robustness across various scales and locations. Although lightweighting typically results in a reduction in model accuracy [24], this issue is addressed through knowledge distillation. The experimental results demonstrate a 9.4% improvement in mAP@50, a 15.6% increase in precision, and a 33% reduction in model parameters. With 24,670 training samples and 6652 validation samples, these improvements not only significantly enhance the model’s overall accuracy but also demonstrate its reliability on large-scale datasets and its practical applicability in real-world scenarios.

### 2.4. Experimental Settings and Training Strategies

First, some tables showing the experimental equipment parameters and experimental training parameters are presented (Table 1, Table 2 and Table 3).

The following are additional key parameter settings. To balance the convergence speed and model stability, the initial learning rate in this experiment was set to 0.01. Various parameters were configured for data augmentation and regularization to enhance the model’s robustness and generalization capabilities. The color augmentation parameters ‘hsv_h’, ‘hsv_s’, and ‘hsv_v’, which adjust hue, saturation, and brightness, were set to 0.015, 0.7, and 0.4, respectively. The geometric transformation parameters ‘degrees’, ‘translate’, ‘scale’, and ‘shear’, used to control rotation, translation, scaling, and shearing, were set to 0.0, 0.1, 0.5, and 0.0, respectively. Flip augmentation was applied with a left–right flip probability, ‘flipud’ and ‘fliplr’, both set to 0.5. These augmentations effectively improved the model’s adaptability to different color conditions, poses, and viewpoints. In the inference and validation stages, the IoU threshold for Non-Maximum Suppression (NMS) was set to 0.7 to reduce overlapping bounding boxes, and the confidence threshold was kept at the default value to ensure detection reliability and minimize missed detections. Through these settings, the model demonstrated high stability and reliability across various conditions, providing strong support for the experimental results.

### 2.5. Evaluation Indicators

In this study, we validate the effectiveness of the proposed method through quantitative metrics such as mAP, Precision (*P*) and Recall (*R*) [25]. mAP is a benchmark metric for the evaluation of target detection algorithms, and we use the DOTA evaluation criteria to calculate it. The precision rate indicates the ability of the model to correctly identify relevant objects, i.e., the proportion of correctly predicted objects among all predictions. Recall measures the ability of the model to identify all relevant objects, i.e., the ratio of the number of true objects predicted by the model to the total number of true objects. *P*, *R* and mAP are calculated as follows:

(7) $$P=\frac{T_{p}}{T_{p}+F_{p}}\times 100\%,$$

(8) $$R=\frac{T_{P}}{T_{P}+F_{N}}\times 100\%,$$

(9) $$AP=\int_{0}^{1}P\left(R\right)dR,$$

(10) $$mAP=\frac{1}{M}\sum_{k=1}^{M}AP\left(K\right),$$

where *T_p_* is true positive, *F_p_* is false positive and *F_n_* is false negative.

## 3. Results

### 3.1. Ablation Experiments

In this comprehensive experimental evaluation, we assessed the performance improvements across multiple iterations of the YOLOv8 model, as detailed in the table. The evaluation focused on several critical metrics, including model size, precision, recall, and average precision, at an IoU threshold of 50% (mAP@50). Additionally, we examined the model’s mAP across IoU thresholds ranging from 50% to 95% (mAP@50:95), providing a more nuanced understanding of its detection capabilities. These metrics collectively offer insights into the trade-offs between model efficiency and accuracy, allowing us to evaluate the impact of architectural modifications, such as lightweight modules and knowledge distillation techniques, on the model’s overall performance (Table 4).

The baseline YOLOv8 model has a size of 5.94 MB, with a precision of 71.3%, recall of 76.7%, mAP@50 of 83.3%, mAP@50:95 of 61.8%, and 8.2 GFLOPs. Incorporating the C2f-STAR module reduced the size to 5.05 MB, while improving the precision to 71.8%, recall to 78.0%, mAP@50 to 84.6%, and mAP@50:95 to 62.8%, with a reduction in GFLOPs to 7.0. Adding the EAM module increased the size slightly to 5.20 MB but further enhanced the recall to 79.5%, mAP@50 to 84.9%, and mAP@50:95 to 63.2%, with GFLOPs at 8.1. The integration of the SSLDH module significantly reduced the model size to 3.98 MB (67% of the baseline size), achieving a precision of 74.5%, recall of 77.7%, mAP@50 of 85.0%, and mAP@50:95 of 63.2%, with GFLOPs reduced to 6.5. After knowledge distillation, the YOLOv8-C2f-STAR-EAM-SSLDH model achieved a precision of 86.9%, recall of 84.1%, mAP@50 of 92.7%, and mAP@50:95 of 70.5%, with the size remaining at 3.98 MB and GFLOPs at 6.5. Compared to the YOLOv8s teacher model (21.4 MB), the distilled model achieved 97.1% of its performance with only 17.6% of its size.

**Figure 8:**
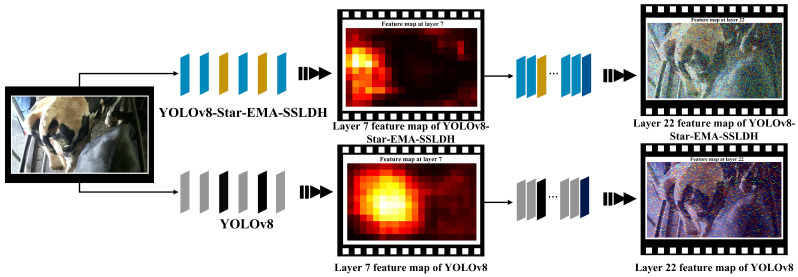
Feature map comparison between original and enhanced YOLOv8 at key layers.

To further evaluate the impact of the newly added modules on model performance, this study conducted a visual analysis of the model’s feature layers, with a focus on two layers significantly influenced by the new modules: Layer 7 and Layer 22. These are shown in Figure 8. Layer 7 is primarily affected by the Star-EMA module, while Layer 22 is positioned near the newly added SSLDH detection head, thereby directly reflecting the effect of the SSLDH module. A comparative analysis of these two feature layers provides a comprehensive view of how the different modules contribute to enhancing the model’s attention distribution and information capture capabilities.

In the feature map of Layer 7, the impact of the Star-EMA module is particularly pronounced. Brighter areas in the feature map indicate regions where the network’s attention is highly focused, while darker areas signify a lower attention distribution. Compared to the feature map of the original YOLOv8 network, the enhanced model shows a more focused attention on the cow’s tail region, indicating that the improvements make the network better suited to the key feature areas required for body condition scoring. This refined attention distribution effectively reduces noise interference and minimizes the capture of irrelevant information, resulting in greater accuracy in this task. In contrast, the attention in the original YOLOv8 network is more dispersed, which likely leads to the capture of extraneous information, impacting the decision accuracy and resulting in decreased performance.

In the feature map of Layer 22, the positive impact of the SSLDH module on the model is further evident. Compared to the original network, the SSLDH module not only effectively reduces the model’s parameter count but also enhances its ability to capture fine-grained image details. Through pixel-based feature visualization, it was observed that the modified model has smaller pixel blocks in Layer 22, indicating that the network can capture more detailed information during feature extraction. This fine-grained information capture improves the model’s discrimination ability in specific regions, contributing to a higher classification accuracy and enabling the model to exhibit greater sensitivity and precision when handling complex images.

The comparative analysis of Layers 7 and 22 validates the role of the Star-EMA module in focusing attention on key feature regions and optimizing attention distribution, while the SSLDH module enhances the granularity of information capture while reducing the model parameters. This analysis clearly demonstrates that the addition of these new modules effectively enhances the overall performance of the model in the BCS task.

### 3.2. Comparative Analysis

To validate the advantages of the improved model, we conducted rigorous benchmarks against several leading algorithms. These include the efficient SSD [26], the widely used YOLOv5, the compact and powerful YOLOv7-tiny [27], the advanced YOLOv9-tiny [28], the distinctive YOLOv10n [29], and the newly introduced YOLOv11n [30]. Additionally, we systematically compared these models by integrating recent academic innovations, such as YOLOv8-CAFM [31] and YOLOv8-C2f-DWR-DRB [32], into the base framework. These algorithms represent the latest advancements in target detection, with their practical advantages and breakthroughs comprehensively demonstrated through multi-dimensional evaluations (Table 5).

The table provides a detailed comparison across multiple dimensions, including model size, precision, recall, mAP@50, and mAP@50:95. Our evaluation includes established models like SSD and recent iterations from the YOLO series, showcasing diverse trade-offs between performance and resource efficiency.

The SSD model demonstrates respectable accuracy (78.6%) and mAP@50 (78.6%), but its large size (118 MB) makes it less suitable for resource-constrained environments. YOLOv7-tiny (11.7 MB) and YOLOv5 (5.01 MB) offer more compact alternatives, but their precision (62.5% and 66.1%) and mAP@50:95 scores (52.0% and 59.0%) indicate moderate detection performance.

More recent iterations, including YOLOv9-tiny, YOLOv10n, and YOLOv11n, provide better balance, with precision scores of 67.0%, 68.8%, and 68.4%, and mAP@50:95 values of 59.8%, 59.3%, and 60.0%. These models maintain compact sizes between 5.2 MB and 5.81 MB, demonstrating their suitability for deployment in low-resource scenarios.

Further improvements are observed in YOLOv8-CAFM (6.60 MB) and YOLOv8-C2f-DWR-DRB (5.70 MB), which achieve higher precision (70.2% and 67.6%) and mAP@50:95 scores (61.1% and 60.3%), indicating better detection capabilities while keeping storage requirements low.

Our proposed BCS-YOLO model stands out, with a compact size of just 3.98 MB while achieving exceptional performance. It delivers the highest precision (86.9%) and recall (84.1%), along with superior mAP@50 (92.7%) and mAP@50:95 (70.5%). These results highlight the effectiveness of the model in improving detection accuracy without compromising on storage and computational efficiency, making it ideal for scenarios with strict hardware and memory constraints.

**Figure 9:**
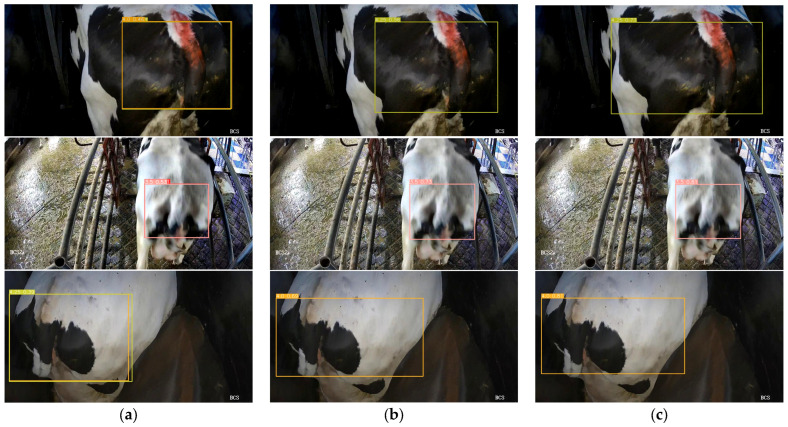
Inference results comparison of different models on the same Image. (**a**) YOLOv8 model inference results. (**b**) YOLOv8-Star-EMA-SSLDH model inference results. (**c**) BCS-YOLO model inference results.

As shown in Figure 9, a comparison of the inference results between the baseline model, the optimized model, and the BCS-YOLO algorithm highlights that the baseline model suffers from issues such as misdetections, overlapping bounding boxes, and low confidence scores, all of which are effectively addressed by BCS-YOLO.

### 3.3. Effects of Different Modules of Attention

In the following experiments, we attempted to replace the EMA module with different modules while keeping the C2f-Star module unchanged. This was done to assess the impact of these modules on the overall network performance. To this end, we evaluated the performance of three different attention mechanisms: the Efficient Local Attention (ELA) [33], the Large Separable Kernel Attention (LSKA) [34], and the Spatial and Channel Reconstruction Convolution (SCConv) [35]; the experimental results are shown in Table 6. The experimental results show that in the task of cow body condition scoring using the C2f-STAR module, when the EMA attention mechanism was added, the performance surpassed that of the ELA, LSKA, and SCConv mechanisms in terms of mAP@0.5.

The network leverages synergistic effects by integrating the C2f-STAR and EMA modules. The EMA module enhances the network’s dimensionality through star operations, thereby providing a richer feature representation space. By restructuring some channels to support batch and group processing, the EMA module promotes the balanced distribution of spatial semantic features within feature groups, which significantly improves feature extraction and integration. This synergy enhances the attention mechanism, leading to a notable improvement in detection accuracy. Additionally, the EMA and SSLDH modules complement each other to further optimize model performance. The SSLDH module refines channel feature processing through star operations, creating a complementary relationship with the EMA module in feature space expansion and detail capture. This combination enhances the network’s capability to handle complex scenes and achieves higher accuracy in target detection and localization.

**Figure 10:**
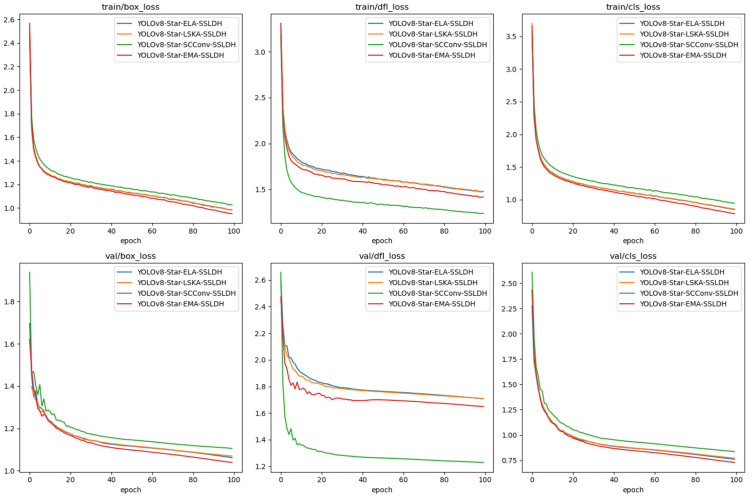
Comparative loss curves of YOLOv8-C2f-Star with different attention modules.

The loss curves clearly demonstrate the EMA module’s significant advantages over ELA, LSKA, and SCConv in the YOLOv8-based BCS framework. As shown in Figure 10, EMA achieves faster and more stable convergence, maintaining consistently lower loss values across training and validation. Its ability to efficiently reconstruct feature channels via star operations enhances spatial and semantic feature alignment, enabling the precise capture of subtle body condition variations like tail curvature and fat reserves.

In training loss curves, EMA excels with rapid, steady declines and the lowest final loss values, reflecting superior localization, reduced boundary ambiguities, and improved classification accuracy. In contrast, SCConv and LSKA show slower convergence and higher losses, highlighting their limitations in handling BCS complexities.

Validation loss curves further confirm EMA’s robustness and generalization. EMA exhibits minimal fluctuations and the lowest losses, indicating strong performance on unseen data. Its smooth val/dfl_loss and val/cls_loss curves emphasize its resistance to overfitting and consistent semantic alignment. By comparison, SCConv shows higher losses and instability, while ELA and LSKA struggle with efficiency and stability.

EMA’s consistent performance across varying conditions underscores its adaptability, where ELA, LSKA, and SCConv fall short due to feature diversity challenges, slower convergence, and redundancy issues. Additionally, the synergy between EMA and SSLDH enhances feature representation and robustness, solidifying EMA as an optimal, efficient component for high-precision BCS tasks.

### 3.4. Effects of Knowledge Distillation

In our experiments, we selected YOLOv8s as the teacher model due to its structural similarity to the student model and its superior performance in object detection tasks. The choice of a structurally aligned teacher ensures better feature alignment during the distillation process, facilitating more effective knowledge transfer. We applied non-destructive performance enhancements to our custom model, YOLOv8-C2f-STAR-EAM-SSLDH, focusing on preserving the integrity of the original architecture while introducing optimizations to boost efficiency and accuracy.

To enhance the knowledge transfer process, we utilized a CWD-based approach that generates soft probability maps by normalizing the activation maps across individual channels. This technique ensures that salient regions within each channel are accurately highlighted, improving the precision of the transferred knowledge. The minimization of the KL divergence between the channel-wise probability distributions of the teacher and student models further ensures a smooth and effective transfer, especially for classification and detection tasks such as cattle body condition scoring, where subtle visual distinctions are crucial for performance.

**Figure 11:**
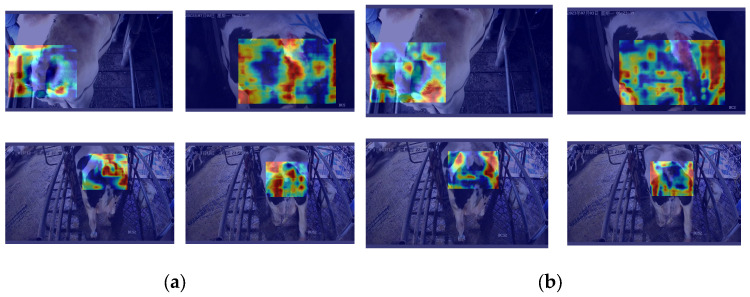
Comparison of attention heat maps: (**a**) before knowledge distillation and (**b**) after knowledge distillation.

Through the distillation process, the student model demonstrated significant improvements in focusing on critical feature regions. The comparison in Figure 11 further reveals the multidimensional impacts and deeper mechanisms of knowledge distillation on the student model. After distillation, the student model exhibited more concentrated and higher-intensity attention in critical regions (e.g., the tailhead area), with feature distributions becoming more compact and boundaries clearer. This improvement was evident not only in the representation of individual feature regions but also in the overall optimization of attention distribution, enabling the model to capture subtle features in local areas (e.g., surface contours and boundary details) more efficiently. Particularly when distinguishing closely scored samples, the distillation strategy significantly enhanced the model’s classification accuracy and discriminative ability.

From a mechanistic perspective, the CWD module played a critical role during distillation by aligning channel-level features and minimizing KL divergence. This allowed the effective transfer of multi-scale salient features from the teacher model to the student model, facilitating deeper feature understanding during multi-layer feature fusion. This mechanism ensured the efficient migration of the teacher model’s feature distribution while strengthening the student model’s ability to comprehensively analyze global and local features. For instance, in key regions such as the tailhead area, the student model accurately captured fine-grained surface variations and critical boundary details, exhibiting superior robustness and adaptability in complex backgrounds and dynamic lighting conditions.

Furthermore, the distillation strategy optimized the allocation of attention to salient regions and reduced redundant feature weights, effectively enhancing the resource utilization efficiency of the student model. While maintaining a lightweight architecture, the student model achieved higher computational efficiency and performance. This capability not only significantly improved the expression of fine-grained features in the body condition scoring task but also enhanced the generalization performance of global features, ensuring the model’s adaptability and stability in diverse real-world scenarios. The impact of the distillation strategy extends beyond performance improvements, offering new possibilities for the scalability of lightweight models. By efficiently compressing and transferring features from the teacher model, the student model not only captured micro-level features in key regions more accurately but also demonstrated stronger generalization capabilities in global feature representation. This provides critical support for the development of lightweight models in practical applications and sets a clear direction for future exploration.

### 3.5. Effects of Dynamic Blurring

**Figure 12:**
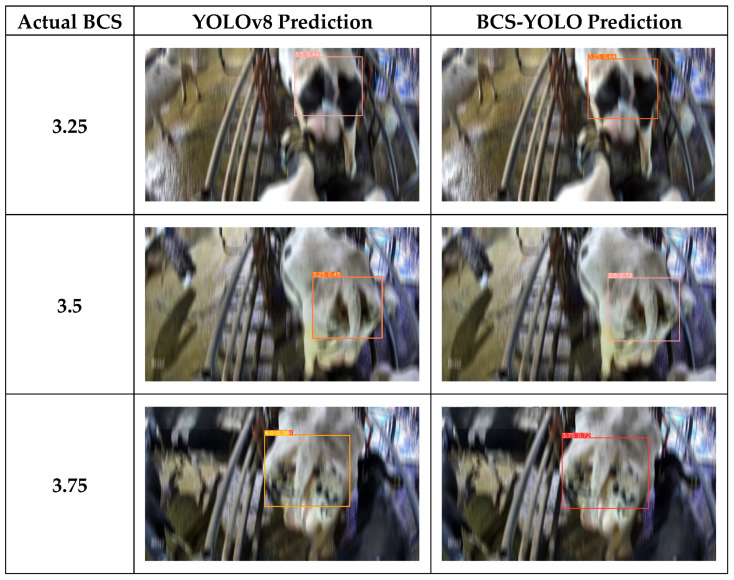

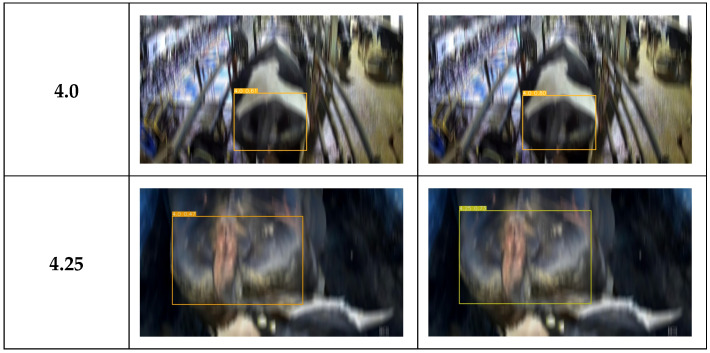
Impact of motion blur on detection.

To evaluate the effectiveness of our module in enhancing robustness and handling dynamic blurring in real-world production environments, we designed a series of specialized experiments. We randomly selected 20% of the validation set images and applied dynamic blurring in the direction of cattle movement, using a blur kernel size of 35. This simulation mimics the image blur resulting from misalignment between the camera shutter speed and cattle motion. Evaluating the model’s performance on these blurred images allows us to test its robustness under non-ideal conditions, particularly in highly dynamic real-world scenarios. A blur kernel size of 35 was chosen to represent the moderate level of motion blur that in typical real environments. This approach not only assesses the model’s accuracy on clear images but also examines its ability to maintain a high detection performance amidst significant visual obstructions. The results will provide insights into the model’s sensitivity to image quality variations and its adaptability to diverse conditions, offering a crucial reference for ensuring its reliable application on real farms. The subsequent section compares the effectiveness of the baseline model with BCS-YOLO (Figure 12):

## 4. Discussion

### 4.1. Interdependencies Between Modules

The collaboration among the Star-EMA, SSLDH, and CWD modules is not merely a simple combination of functionalities but rather a highly interactive optimization loop that delivers a precise, lightweight, and efficient solution for dairy cow body condition scoring. These modules complement each other in feature extraction, fusion, and knowledge transfer, forming a dynamic dependency and feedback mechanism that effectively addresses the performance limitations caused by feature loss and computational constraints in traditional lightweight designs.

The Star-EMA module employs multi-scale attention mechanisms to focus on extracting critical regional features, such as the tailhead area, enhancing the model’s ability to capture fine-grained details. However, its limitations in global feature representation necessitate the multi-scale fusion capabilities of the SSLDH module for supplementation. The quality of the local features extracted by Star-EMA directly influences the fusion efficiency of SSLDH, while the global feature integration provided by SSLDH, in turn, complements the local feature extraction of Star-EMA. This bidirectional dependency forms the foundation for effective feature fusion.

The SSLDH module reduces the parameter count through its lightweight design and shared convolutional kernels, all while refining the pixel block granularity to enhance feature detail representation, enabling more precise classification and output. However, such a lightweight design inevitably risks the loss of fine-grained details. To mitigate this, the CWD module utilizes knowledge transfer to align and integrate the outputs of Star-EMA and SSLDH into the student model, compensating for feature loss while improving robustness and generalization. Acting as an “information lubricant”, CWD strengthens the collaboration between Star-EMA and SSLDH, facilitating more efficient feature absorption and integration during knowledge transfer. CWD serves not only as the product of this collaboration but also as its driving force.

This collaboration operates as a dynamic feedback loop: the detail-capturing ability of the Star-EMA module is enhanced through SSLDH’s global feature fusion, while the trade-offs of SSLDH’s lightweight design are effectively balanced by the knowledge transfer capabilities of CWD. This feedback ensures a continuous cycle of information flow and feature optimization. For instance, Star-EMA’s ability to focus on critical regions is further amplified by SSLDH’s multi-scale fusion, while CWD compensates for the feature loss inherent in lightweight design, thereby maximizing the overall efficiency of the collaborative process.

This collaborative module strategy offers a novel approach to balancing performance and efficiency, advancing the application of lightweight deep learning models in agricultural intelligence. Future research could explore more advanced collaborative mechanisms and diverse use cases to fully realize the potential of this framework.

### 4.2. BCS-YOLO Limitations and Dataset Expansion

Due to the limitations of the training dataset, the BCS-YOLO model exhibits certain constraints. The current dataset primarily focuses on body condition scores ranging from 3.25 to 4.25, reflecting the typical state of lactating cows in large-scale farming environments. Scores between 1.0 and 2.0 are rarely observed in industrial-scale farms, as such cows generally exhibit severe health issues, while scores between 4.5 and 5.0 represent high-risk groups that are similarly excluded. This restricted scoring range may limit the model’s applicability under extreme BCS conditions, as its performance has not been validated for cows with scores below 3.25 or above 4.25 [36].

Additionally, while the dataset classifies BCS with increments of 0.25, this granularity may be insufficient to capture dynamic fluctuations in body condition scores, particularly during lactation, where scores typically range from 2.2 to 4.0, with an average of 3.29 ± 0.25. Such fluctuations can impact the model’s ability to accurately assess subtle changes in health status and productivity.

To address these limitations, future research should focus on expanding the dataset’s scoring range, particularly by incorporating data that represent the full spectrum of scores from 1 to 5 and ensuring the adequate representation of extreme conditions. Furthermore, the dataset should include a greater diversity of cow breeds and farming environments to enhance the model’s robustness and generalizability. These improvements would not only clarify the model’s applicability across various scenarios but also support further optimization to better meet practical demands.

### 4.3. BCS-YOLO in Dairy Cow Health Management

While the lightweight design of BCS-YOLO substantially enhances the efficiency of model deployment, its primary significance lies in its potential to improve individual dairy cow health management. Animal health and welfare are foundational to the sustainability of modern livestock farming. As sentient beings, the health of dairy cows not only affects productivity but also plays a pivotal role in the transmission of diseases within herds and in broader public health concerns. Body condition scoring, a critical metric for evaluating nutritional status and overall health, is indispensable in precision livestock management. From a clinical veterinary perspective, BCS is considered an essential component of routine health evaluations, serving as an early diagnostic tool for identifying potential health issues such as metabolic disorders, ketosis, and fatty liver. These conditions can have profound implications for both individual animal welfare and the overall management of the herd. By utilizing a contactless, automated scoring system, farmers and veterinarians can efficiently monitor the body condition of individual cows, facilitating the early detection of health problems. This proactive approach enables timely veterinary intervention, which is crucial for preventing the spread of diseases, improving animal welfare, and enhancing farm productivity. BCS-based health monitoring is pivotal for advancing the implementation of refined management practices in large-scale farming operations, reducing reliance on subjective assessments, and ensuring a more precise and compassionate approach to animal care.

### 4.4. Future Work

This study has established a solid foundation for the intelligent application of body condition scoring in dairy cows. It underscores the feasibility of developing a cost-effective system using standard network cameras and compact industrial computers to deploy neural network models. Looking ahead, several key directions can be pursued to further refine and expand the system’s functionality.

(1) Expansion and Optimization of the Dataset

The current dataset lacks comprehensive coverage of different growth stages and diverse rearing environments. To address this gap, future research should collaborate with veterinarians and farms to gather real-world information related to body condition scoring, in order to validate the system’s adaptability and effectiveness across different environments. Future research should expand the dataset to include calves, heifers, and lactating cows, as these stages exhibit notable differences in body size, fat distribution, and physical structure. Incorporating such data will improve the model’s ability to capture body condition changes and enhance its applicability across growth phases. Additionally, datasets from extreme environments, such as high-temperature and high-humidity or cold climates, are essential. For instance, heat stress can reduce feed intake and deteriorate animals’ body condition, while cold climates may prompt the mobilization of fat reserves to maintain body temperature. Including these environmental datasets will enhance the model’s adaptability to varying stress conditions, improving its robustness and stability in complex agricultural scenarios.

(2) Further Optimization of Model Structure

Although the proposed lightweight model has achieved significant reductions in parameter size and computational complexity, there remains potential for further optimization. Future work could explore the use of neural architecture search (NAS) to automatically design more efficient model structures tailored to BCS tasks. Additionally, incorporating model pruning techniques to optimize network layer design can further reduce redundant computations while maintaining model performance. These advancements will improve the model’s efficiency, enabling it to perform effectively in resource-constrained environments and providing more cost-effective solutions for real-world applications.

(3) Development of a Longitudinal BCS Database

Future research could focus on creating a longitudinal BCS database that integrates body condition scores with visual ID recognition, enabling the tracking of individual cows’ historical condition changes. To enhance the accuracy and practicality of this database, close collaboration with veterinary professionals will be essential in future research. By integrating clinical health data, this approach will provide stronger support for the comprehensive assessment of dairy cow health. Combining these data, the system will not only offer more precise body condition evaluations but also provide critical information for early intervention and health management, thereby facilitating the early identification of potential health issues and further optimizing herd management strategies. This approach will enhance health management and production optimization, advancing precision livestock management and supporting the modernization of dairy farming.

## 5. Conclusions

In this study, we introduce BCS-YOLO, a contactless cattle body condition scoring model tailored to large-scale dairy farms. BCS-YOLO addresses the limitations of traditional body condition scoring methods, which are often time-consuming, subjective, and challenging to scale, by optimizing its architecture and incorporating a lightweight backbone network, an efficient neck module, and a highly effective detection head. Central to this optimization are two key innovations: the Star-EMA and SSLDH modules. The Star-EMA module effectively combines the star operation with the EMA attention mechanism, significantly expanding the feature space dimensions. This enhancement allows the model to more accurately capture subtle variations in the cow’s body shape, thereby improving the classification accuracy and robustness. Conversely, the SSLDH module employs a combination of star operations and GroupNorm convolution to reduce the number of parameters while preserving the computational efficiency and feature representation. This design enhances the model’s flexibility and efficiency for practical deployment.

Additionally, we employed the CWD-based feature knowledge distillation technique as part of the overall optimization strategy. This approach further enhances the feature extraction accuracy and computational performance by effectively complementing the feature processing methods of Star-EMA and SSLDH. It achieves a significant improvement in BCS-YOLO’s scoring accuracy without increasing the model’s complexity. This strategy not only reduces training costs but also accelerates model convergence, making BCS-YOLO well suited for environments with limited computational resources.

Through comprehensive experimental validation, BCS-YOLO has demonstrated outstanding performance in body condition scoring tasks. Despite maintaining a lightweight model, BCS-YOLO achieves significant improvements in key metrics, including precision, recall, and mAP. Specifically, compared to the baseline model, BCS-YOLO increases mAP by nearly 9.4 percentage points while significantly reducing the model size. Additionally, mAP@50:95 shows a corresponding improvement, underscoring its superiority and practicality in complex application scenarios. More importantly, BCS-YOLO not only optimizes production management processes but also advances individual-based health monitoring, enabling farmers to assess changes in cow body conditions more conveniently and promptly identify potential health issues. This capability is critical for ensuring animal welfare, reducing the risk of disease transmission, and enhancing farm sustainability. As BCS-YO is adopted in broader real-world applications, it is expected to further improve dairy cattle health management and precision livestock practices, driving modern agriculture toward greater efficiency and sustainability.

## Figures and Tables

**Table 1:** Specifications of the experimental platform.

Parameters	Configuration
CPU	Intel^®^ Core^TM^ i5-13600KF (Santa Clara, CA, USA)
GPU	NVIDIA GeForce RTX3070 (Santa Clara, CA, USA)
Compilers	Python 3.11
Network construction method	Pytorch 2.0.0
Running memory	16 GB
Operational platform	Windows 10

**Table 2:** Key training parameters.

Parameters	Setup
Epochs	100
Batch size	16
Works	4
Input image size	640
Optimizer	SGD

**Table 3:** Training and knowledge distillation hyperparameters.

Hyperparameters	Configuration
Epochs	100
Batch size	16
Knowledge Distillation Loss Type	Feature
Knowledge Distillation Loss Decay	Constant
Teacher Knowledge Distillation Layers	12, 15, 18, 21
Student Knowledge Distillation Layers	12, 15, 18, 21

**Table 4:** Ablation study results of YOLOv8 variants.

Model	Size (MB)	Precision/%	Recall/%	mAP@50/%	mAP@50:95/%	GFLOPs
YOLOv8 (baseline)	5.94	71.3	76.7	83.3	61.8	8.2
YOLOv8-C2f-Star	5.05	71.8	78.0	84.6	62.8	7.0
YOLOv8-C2f-Star-EAM	5.20	71.3	79.5	84.9	63.2	8.1
YOLOv8-C2f-Star-EAM-SSLDH	3.98	74.5	77.7	85.0	63.2	6.5
YOLOv8-C2f-Star-EMA-SSLDH (Distilled)	3.98	86.9	84.1	92.7	70.5	6.5
YOLOv8s (Teacher mold)	21.4	89.9	88.6	95.5	74.8	28.7

**Table 5:** Comparison of model performance across different architectures.

Model	Size (MB)	Precision/%	Recall/%	mAP@50/%	mAP@50:95/%	GFLOPs
SSD	118	78.6	70.5	78.6	53.6	33.82
YOLOv7-tiny	11.70	62.5	73.1	75.3	52.0	13.2
YOLOv11n	5.20	68.4	76.4	81.8	60.0	6.3
YOLOv10n	5.47	68.8	76.8	81.9	59.3	9.1
YOLOv9-tiny	5.81	67.0	77.9	81.4	59.8	11.0
YOLOv5	5.01	66.1	76.3	80.6	59.0	7.2
YOLOv8-CAFM	6.60	70.2	77.2	83.8	61.1	8.5
YOLOv8-C2f-DWR-DRB	5.70	67.6	77.5	81.6	60.3	8.0
YOLOv8-C2f-Star-EAM-SSLDH	3.98	74.5	77.7	85.0	63.2	6.5
BCS-YOLO	3.98	86.9	84.1	92.7	70.5	6.5

**Table 6:** Experimental results of different attention modules with YOLOv8-C2f-Star.

Model	Size (MB)	Precision/%	Recall/%	mAP@50/%	mAP@50:95/%
YOLOv8-C2f-Star-ELA-SSLDH	4.67	69.7	77.5	82.8	61.1
YOLOv8-C2f-Star-LSKA-SSLDH	4.72	70.7	76.2	82.4	60.9
YOLOv8-C2f-Star-SCConv-SSLDH	5.34	69.1	74.7	80.9	58.6
YOLOv8-C2f-Star-EAM-SSLDH	3.98	74.5	77.7	85.0	63.2

## Data Availability

The data presented in this study are available upon https://www.scidb.cn/en/detail?dataSetId=16b8bdaf31ee4c8b9891fc7e9df6e41c&version=V2 (accessed on 23 April 2024).
